# Typhoid Conjugate Vaccines: Advancing the Research and Public Health Agendas

**DOI:** 10.1093/infdis/jiab449

**Published:** 2021-09-16

**Authors:** Megan Birkhold, Aziza Mwisongo, Andrew J Pollard, Kathleen M Neuzil

**Affiliations:** 1 Center for Vaccine Development and Global Health, University of Maryland School of Medicine, Baltimore, Maryland, USA; 2 Center for Vaccine Innovation and Access, PATH , Seattle, Washington, USA; 3 Oxford Vaccine Group, Department of Paediatrics, University of Oxford, Oxford, United Kingdom; 4 National Institute for Health Research Oxford Biomedical Research Centre, Oxford, United Kingdom

**Keywords:** Africa, Asia, *Salmonella* Typhi, typhoid conjugate vaccines, typhoid fever, TyVAC

## Abstract

The disease burden of typhoid fever remains high in endemic areas in Asia and Africa, especially in children. Recent clinical trials conducted by the Typhoid Vaccine Acceleration Consortium show typhoid conjugate vaccine (TCV) to be safe, immunogenic, and efficacious at preventing blood culture-confirmed typhoid fever in African and Asian children. Pakistan, Liberia, and Zimbabwe recently introduced TCV through campaigns and routine childhood immunizations, providing protection for this vulnerable population. It is essential to continue this momentum while simultaneously filling data gaps—including typhoid complications—to inform decision-making on TCV introduction. A multidisciplinary approach including surveillance, water, sanitation, and hygiene investments, and large-scale TCV introduction is needed to decrease the burden and mortality of typhoid fever.

*Salmonella enterica* serovar Typhi (*S.* Typhi) is the causative agent of typhoid fever, a disease characterized by fever, malaise, anorexia, abdominal pain, and other gastrointestinal symptoms. Due to improvements in water and sanitation infrastructure, typhoid fever has mostly been eliminated in high-income countries [1]. However, typhoid fever continues to disproportionally affect low- and middle-income countries, with about 9 million cases and more than 110 000 attributable deaths per year globally [2]. Complications can arise when this infection goes untreated, or is improperly treated, leading to septic shock, gastrointestinal hemorrhage, or intestinal perforation resulting in an estimated 1%–4% fatality with, and 10%–20% without, treatment [3, 4].

Disease burden is especially high in endemic areas of sub-Saharan Africa, South and Southeast Asia, and Oceania, with children particularly vulnerable [5–7]. Increasingly, multidrug resistant and extensively drug resistant (XDR) strains of *S.* Typhi have been identified in endemic areas [8]. If these strains continue to spread globally, current antibiotic treatments will become increasingly ineffective, leading to an increased burden on the health care system, a rise in severe complications, and, ultimately, in mortality rates.

In this article, we provide an overview of typhoid vaccines, review the policy timeline for typhoid conjugate vaccine (TCV), and present data generated from recent clinical trials in endemic countries. The trial results demonstrate TCV is safe, immunogenic, and efficacious in Africa and Asia, even in children younger than 2 years. These data inform decision-making on TCV introduction through campaigns and incorporation into a country’s expanded program on immunization (EPI), a critical avenue to distribute life-saving vaccines.

## TYPHOID VACCINES

There are currently 3 licensed typhoid vaccines: TCV, live attenuated Ty21a, and a Vi capsular polysaccharide vaccine (ViCPS). The World Health Organization (WHO) has recommended typhoid vaccines in endemic countries since 2008. However, uptake of Ty21a and ViCPS has been low due to the number of required doses, duration of protection, lack of Gavi, the Vaccine Alliance (Gavi) funding, and because neither vaccine is approved for children younger than 2 years [9, 10]. For additional information, Sahastrabuddhe and Saluja [11] provide a detailed discussion of current and future typhoid vaccines and in this supplement, Shakya, Neuzil, and Pollard [10] discuss typhoid and paratyphoid vaccines in endemic settings.

TCVs are advantageous because they overcome some of the limitations that inhibited uptake of prior vaccines. In particular, TCVs are suitable for children younger than 2 years, which allows for inclusion in routine childhood immunization programs. Typbar TCV, manufactured by Bharat Biotech Ltd, was prequalified by WHO in late 2017 and recommended by WHO in 2018. Typbar TCV consists of the Vi polysaccharide (Vi) of *S.* Typhi conjugated to a tetanus toxoid (TT) carrier protein [9]. It is administered in a single dose and approved for children 6 months of age and older. In December 2020, TYPHIBEV, manufactured by Biological E, was prequalified by WHO. Similar to Typbar TCV, it is administered in a single dose and approved for children older than 6 months; it differs, however, in that the Vi polysaccharide is conjugated to the CRM197 protein.

## THE TYPHOID VACCINE ACCELERATION CONSORTIUM

In 2016, the Typhoid Vaccine Acceleration Consortium (TyVAC) was funded by the Bill and Melinda Gates Foundation with the goal to accelerate introduction of TCVs into low-resource countries eligible for funding from Gavi. TyVAC collaborates with local and global stakeholders to facilitate TCV access and optimize impact in the most marginalized communities. TyVAC supports an integrated approach to typhoid prevention and control, with TCVs and improved water, sanitation, and hygiene (WASH), to reduce typhoid fever burden. A major aim of TyVAC was to collate existing data and generate new evidence—through clinical trials in Bangladesh, Burkina Faso, Malawi, and Nepal—on TCV safety, effectiveness, and coadministration, as well as disease burden, drug resistance, and cost-effectiveness, to inform national and global decision-makers.

## PREQUALIFICATION OF TYPHOID CONJUGATE VACCINES

**Figure 1.**
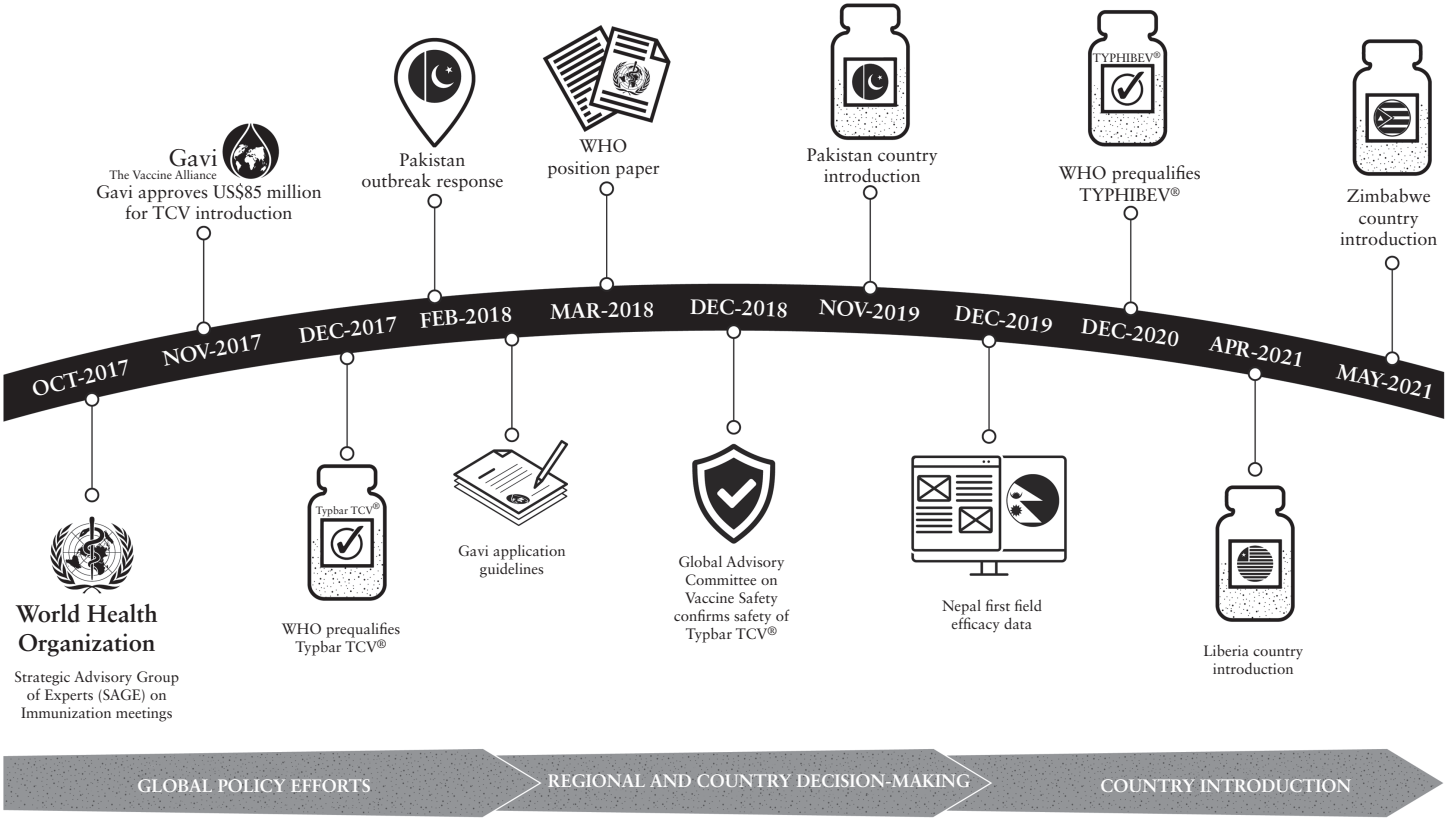
Timeline of TCV policy and introduction milestones. Abbreviations: Gavi, Gavi, The Vaccine Alliance; TCV, typhoid conjugate vaccine; WHO, World Health Organization.

In 2018, due to the continued high burden of typhoid fever, increasing antimicrobial resistance (AMR), and with input from the Strategic Advisory Group of Experts (SAGE), WHO recommended TCV for children 6 months of age and older along with TCV catch-up vaccination for children younger than 16 years in endemic countries (Figure 1) [9]. The WHO outlined a research agenda that included robust surveillance, data on coadministration of TCV with routine childhood immunizations, duration of protection of TCV after a single dose, the potential timing and need for a booster, and a better understanding of tetanus immunity induced by the TT carrier protein of the Vi-TT conjugate vaccine [9].

## TYVAC CLINICAL TRIALS

At the start of TyVAC, there were no efficacy data for Typbar TCV, the first WHO-prequalified TCV. A phase 3 study in India demonstrated safety and immunogenicity of TCV in healthy infants, children, and adults [12]. The vaccine was licensed in India, based on these safety and immunogenicity data, as a single-dose vaccine in children aged 6 months and older.

In addition to the immunogenicity studies in India, controlled human infection studies in adults in the United Kingdom provided evidence for the efficacy of TCV and informed the SAGE recommendation [13]. However, the need for pediatric efficacy trials in endemic areas was identified as a priority to catalyze vaccine introduction decisions. High-quality clinical trials are expensive and labor intensive and therefore only a limited number could be conducted in a timely fashion. Thus, it was important to conduct trials in diverse epidemiologic settings and populations to serve as prototypes so data from these trials could be extrapolated to other areas. Logistical considerations were also paramount. The trials needed to be conducted in settings with enough disease burden to establish efficacy with reasonable sample sizes and in realistic time frames. Because typhoid fever may be confused with other febrile infections, the ability to conduct robust and comprehensive blood cultures was a necessary prerequisite for a TyVAC clinical trial site. A prior typhoid surveillance study, the Strategic Typhoid Alliance across Africa and Asia (STRATAA) study, had sites with necessary infrastructure and capacity and, therefore, were ideal sites for the conduct of efficacy trials [14]. The strengths and weaknesses of different study designs were assessed with the goal to design unbiased, ethical, controlled trials appropriate for each study site [15]. Studies were designed to generate efficacy data across broad pediatric age groups to measure indirect protection conferred by TCVs and to include both specific (blood culture-confirmed) and serious (hospitalizations and complications) end points.

**Figure 2.**
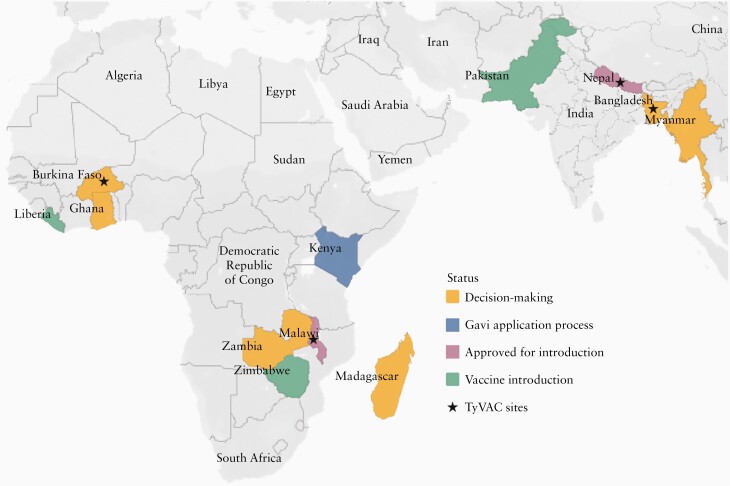
Map of TyVAC trial sites and country introduction status. Abbreviation: Gavi, Gavi, The Vaccine Alliance; TyVAC, Typhoid Vaccine Acceleration Consortium.

Ultimately, the decision was made to conduct individually randomized phase 3 trials in Malawi and Nepal and a phase 3 cluster-randomized trial in Bangladesh (Figure 2) [16–18]. The primary objective of the individually randomized trials was to determine the efficacy of TCV in reducing rates of symptomatic, blood culture-confirmed *S.* Typhi infection [16, 17]. Safety and immunogenicity were important secondary objectives. The Bangladesh study design allowed for estimates of overall and direct effects and prespecified blood culture-confirmed typhoid fever as the primary end point measure [18].

In parallel to the efficacy studies, additional studies were conducted based on the WHO research priorities [9]. In Burkina Faso, a phase 2 individually randomized trial was designed to assess safety and immunogenicity of TCV when coadministered with vaccines routinely given at 9 months (measles-rubella and yellow fever vaccines) and 15 months (meningococcal A vaccine) of age [19]. One- versus 2-dose immunogenicity studies in Nepal (among children aged 9 to 15 months) and Malawi (among HIV-exposed and unexposed children at 9 months of age) were likewise conducted. Table 1 and Table 2 show details of the TyVAC trials.

**Table 1.** Typbar Typhoid Conjugate Vaccine (TCV) Evaluation in Field Settings to Inform Country-Level Decision-Making: Studies of Safety, Immunogenicity, and Efficacy/Effectiveness of Single-Dose TCV

Country	Design	Control Vaccine	Age	Study Period (Participant Follow-up)	No. Vaccinated	Cases/100 00 Person-Years, Control Group	Vaccine Efficacy BC-Confirmed Typhoid, %
Nepal	Individually randomized	Meningococcal serogroup A conjugate	9 mo to <16 y	Nov 2017–Jan 2020 (2 y)	20 019	337	81.6^a^
Malawi	Individually randomized	Meningococcal serogroup A conjugate	9 mo to <13 y	Feb 2018–Sep 2021 (3 y)	28 052	260	83.7^b^
Bangladesh	Cluster-randomized	Live attenuated Japanese encephalitis	9 mo to <16 y	Apr 2018–May 2020 (2 y)	67 395	635	85.0^c^

^a^Blood-culture (BC) confirmed typhoid fever after 1 year of follow-up. Per protocol analysis.

^b^Blood-culture confirmed typhoid fever after 18–24 months of follow-up. Per protocol analysis.

^c^Total effectiveness.

**Table 2.** Typbar Typhoid Conjugate Vaccine Evaluation in Field Settings to Inform Country-Level Decision-Making: Reactogenicity, Immunogenicity, and Coadministration Studies

Country	Design	Control Vaccine	Age	Study Period	No. Enrolled	Coadministered Vaccines	Other
Burkina Faso	Individually randomized	Inactivated poliovirus	9–11 mo	Nov 2018–Aug 2019	100	Measles, rubella, yellow fever	…
Burkina Faso	Individually randomized	Inactivated poliovirus	15–23 mo	Nov 2018–Aug 2019	150	Meningococcal serogroup A conjugate, measles, rubella^a^	…
Nepal	Individually randomized	Meningococcal serogroup A conjugate	9–12 mo	Nov 2017–ongoing^b^	100		1 vs 2 doses
Malawi	Individually randomized	Meningococcal serogroup A conjugate	9 mo	Mar 2021–ongoing^b^	100	Measles, rubella	HIV-exposed, 1 vs 2 doses

^a^Measles rubella second dose coadministered; no assessment of immunogenicity.

^b^Interrupted by COVID-19 pandemic.

### Results

The first efficacy results from Nepal showed a TCV efficacy against blood culture-confirmed typhoid fever of 81.6% after 1 year [20]. When the analysis was restricted to the WHO clinical diagnosis of typhoid fever, those presenting with at least 3 days of fever, the efficacy estimate increased to 85.1% [20]. Similar vaccine efficacy was observed in Malawi, with an efficacy estimate of 80.7% (intention-to-treat population) and 83.7% (per protocol population) against blood culture-confirmed typhoid fever among children 9 months through 12 years of age, after 18 months of study follow-up (Table 1) [21]. In a cluster-randomized trial in Dhaka, Bangladesh total effectiveness of TCV was 85% among vaccinated children aged 9 months to 15 years [22]. Importantly, total effectiveness remained high across age groups, including among children younger than 2 years of age, in whom total effectiveness was 81% [22]. High efficacy after a single dose, and the consistency of results in different settings and age groups, supports broad use of TCV (Table 1).

### Safety and Immunogenicity

The immunogenicity, reactogenicity, and safety profile of TCV were important end points of the trials. When compared to the control vaccine, Vi-immunoglobulin G (IgG) geometric mean titers were significantly increased (*P* < .001) for TCV in Bangladesh, Malawi, and Nepal in all age groups, confirming the high immunogenicity of TCV reported in India [20–22]. Results from Burkina Faso on coadministration of TCV with yellow fever and measles-rubella vaccines at 9 months of age and group A meningococcal conjugate vaccine at 15 months old showed comparable TCV antibody responses in the countries where efficacy has been demonstrated (Table 2), again confirming the consistency in the performance of this vaccine across diverse settings. For all vaccines tested—yellow fever, measles-rubella, and group A meningococcal conjugate—antibody responses were similar in the groups receiving concomitant TCV as compared to the groups not receiving concomitant TCV [23, 24]. This demonstrates safe coadministration is possible, which facilitates incorporation into routine immunization schedules. Additionally, postvaccination anti-tetanus IgG antibody titers exceeded thresholds for long-term immunity in all but 1 participant, supporting an added benefit of TCV, increasing long-term immunity to tetanus [23].

The safety profile of TCV in these 4 trials was consistent with those of a phase 3 trial in India, where no serious vaccine-attributable adverse events were observed [12]. In each trial, similar adverse events were observed in the TCV and control vaccine groups (group A meningococcal conjugate vaccine in Malawi and Nepal and Japanese encephalitis vaccine in Bangladesh), indicating an acceptable vaccine safety and tolerability profile. The Global Advisory Committee on Vaccine Safety (GACVS), after review of relevant data, concluded “the safety profile of the Typbar TCV vaccine is reassuring, and no signals of serious adverse events were presented” (Figure 1) [25]. GACVS recommended continued safety monitoring in countries that incorporate TCV into their routine immunization programs or that implement mass vaccination campaigns.

### Additional Clinical Trial Outcomes

A number of exploratory outcomes, including number and duration of all-cause or typhoid-specific hospitalizations, typhoid complications (eg, perforations or gastrointestinal hemorrhage), antibiotic usage, and outpatient visits were prestated in the trial protocols. Results on age-specific efficacy, duration of protection, and additional results on safety and immunogenicity are forthcoming and will allow a comprehensive assessment of the public health impact of TCVs. The vital information collected from these clinical trials can then be used to further guide TCV introduction in endemic areas.

## INTRODUCTION OF TCVS IN ENDEMIC AREAS

Based on the WHO SAGE recommendations for TCV introduction, Gavi announced support for TCVs in November 2017 and allocated $85 million of funding support for Gavi-eligible countries. In early 2019, as a response to a typhoid fever outbreak, the Zimbabwe Ministry of Health carried out an emergency mass TCV campaign through the WHO and subsidized with Gavi funding. This was the first programmatic use of TCV in Africa and resulted in decreased typhoid cases [26]. In November 2019, Pakistan became the first country to introduce TCV into its routine immunization schedule, a decision driven by an XDR typhoid outbreak. Pakistan used a phased approach, starting with a mass TCV vaccination campaign in Sindh Province then moving to Islamabad and Punjab Province, to ultimately vaccinate about 23 million children with TCV. Results from the outbreak response in Pakistan show TCV was 95% effective against blood-culture confirmed typhoid fever and 97% effective again XDR typhoid fever [27]. Liberia was the first African country to introduce TCV in a nonoutbreak setting, starting with a week-long campaign in April 2021 for children aged 9 months to younger than 15 years, followed by integration into the country’s routine immunization program. In May 2021, Zimbabwe began its large national TCV campaign, with the goal to vaccinate 6.2 million children aged 9 months to younger than 15 years [28]. Following this campaign, through their EPI, TCV will be available for administration alongside the measles vaccine for all 9-month-old infants [28].

With Gavi funding, additional countries can join Pakistan, Liberia, and Zimbabwe and introduce TCV, helping to protect children from this deadly infection. Numerous countries in both Africa and Asia are currently in various stages of the application and introduction process (Figure 2). TyVAC and its partners will continue to support introduction efforts in these countries, with the ultimate goal of reducing the global burden of typhoid, especially in endemic areas. These introductions provide additional learning opportunities to inform programmatic use of TCV.

## CONTINUING THE MOMENTUM AND FILLING THE KNOWLEDGE GAPS

The coronavirus disease 2019 (COVID-19) pandemic interrupted vaccine campaigns and slowed the momentum of new vaccine introductions, including TCV. The ongoing work in Pakistan, Liberia, and Zimbabwe is a testament to the political will of those countries in prioritizing child health; however, 3 countries are not enough. Data are an essential component to drive new vaccine introductions. Unfortunately, many countries in Asia and Africa lack adequate data on typhoid fever incidence, complications, AMR, and morbidity and mortality rates to inform TCV introduction decisions. Additionally, even in countries with surveillance programs, many are restricted to tertiary hospitals and urban centers, leaving rural and remote areas with significant knowledge gaps.

Recent complication and mortality reviews have highlighted the limited data available in endemic regions [3, 29]. A better understanding of typhoid complications may be one way to fill knowledge gaps and inform decisions on vaccine introduction. Typhoid intestinal perforations, for example, have pathognomic surgical features that may serve as an indicator of typhoid disease burden in poor or rural settings with limited blood culture capability. In Malawi, monthly counts of intestinal perforations tracked with seasonality of typhoid fever cases in a study conducted between January 2008 and June 2015 [30]. While the incidence of typhoid intestinal perforations is unknown, when they do occur the associated mortality is unacceptably high; a recent systematic review found a median case fatality ratio of 20% among African children presenting with typhoid intestinal perforations [3]. Establishing partnerships with surgeons and surgical departments in areas where blood and other sterile site cultures are not available or feasible, could allow for comparisons of typhoid perforation cases before and after TCV introduction.

## CONCLUSION

Typhoid fever, and its complications, continue to cause significant morbidity and mortality in low-resource settings. While integrated solutions including access to health care and improvements in WASH infrastructure are needed, these take time and a substantial investment, leaving populations, especially children, vulnerable to this disease. Introduction of TCV is a viable option that would reduce mortality, combat AMR, and decrease the overall disease burden. A multidisciplinary, collaborative approach is necessary for combating typhoid fever.

## Notes

***Acknowledgment.*** The authors thank Dr Anthony Marfin and Leslie Jamka for input and review of the manuscript.

***Financial support.*** This work was supported by the Bill and Melinda Gates Foundation (grant number OPP1151153); and the National Institutes of Health (grant number T32 DK067872 to M. B.).

***Potential conflicts of interest.*** K. M. N. and A. J. P. are members of the World Health Organization Strategic Advisory Group of Experts on Immunization. A. J. P. is Chair of the UK Department of Health and Social Care’s Joint Committee on Vaccination and Immunisation. A. J. P. institution, The University of Oxford, has entered into a partnership with AstraZeneca on coronavirus vaccine development. All other authors report no potential conflicts.

***Data availability***. Data are currently unavailable because the studies are ongoing.

Presented in part: 15th Asian Conference on Diarrhoeal Disease and Nutrition, January 2020, Dhaka, Bangladesh. Under the grant conditions of the Bill and Melinda Gates Foundation, a Creative Commons Attribution 4.0 Generic License has already been assigned to the Author Accepted Manuscript version arising from this submission.
